# The Scientific Contribution of the Kaniadakis Entropy to Nuclear Reactor Physics: A Brief Review

**DOI:** 10.3390/e25030478

**Published:** 2023-03-09

**Authors:** Aquilino Senra Martinez, Willian Vieira de Abreu

**Affiliations:** Instituto Alberto Luiz Coimbra de Pós-Graduação e Pesquisa em Engenharia (COPPE/UFRJ), Programa de Engenharia Nuclear (PEN), Universidade Federal do Rio de Janeiro, Rio de Janeiro 21941-914, Brazil

**Keywords:** Kaniadakis entropy, Doppler broadening function, nuclear reactor physics

## Abstract

In nuclear reactors, tracking the loss and production of neutrons is crucial for the safe operation of such devices. In this regard, the microscopic cross section with the Doppler broadening function is a way to represent the thermal agitation movement in a reactor core. This function usually considers the Maxwell–Boltzmann statistics for the velocity distribution. However, this distribution cannot be applied on every occasion, i.e., in conditions outside the thermal equilibrium. In order to overcome this potential limitation, Kaniadakis entropy has been used over the last seven years to generate generalised nuclear data. This short review article summarises what has been conducted so far and what has to be conducted yet.

## 1. Introduction

In 2001, Giorgio Kaniadakis presented [1] a new one-parameter deformation for the exponential function, which describes power-law asymptotic behaviour. He did this in order to obtain a novel distribution that generalises the Maxwell–Boltzmann (MB) one. This new deformed exponential is provided by:

(1) expκ(x)≡(1+κ2x2+κx)1κ,

where the κ parameter represents the level of deformation concerning the standard exponential. Hence, when κ→0, the deformed exponential reduces to exp(x). 

The $\exp_{\kappa }\left(x\right)$, function obeys the following condition:

(2) $$\exp_{\kappa }\left(x\right)\exp_{\kappa }\left(-x\right)=1.$$

By considering the deformed exponential function, one can obtain a new statistical distribution, starting from a particle system in the velocity space and postulating a generalised density of entropy, given by [1,2]:

(3) $$\sigma_{\kappa }\left(f\right)=-\int df\ln_{\left\{\kappa \right\}}\left(\alpha f\right).$$

With that, after some calculations, one can arrive at the following distribution function [1,2,3]:

(4) $$f_{\kappa }\left(V,T\right)=A\left(\kappa \right)\exp_{\kappa }\left(-\frac{MV^{2}}{2k_{B}T}\right),$$

where in nuclear reactor physics terms, *M* is the nucleus mass, *V* is the velocity of the target nucleus, $k_{B}$ the Boltzmann constant, T is the temperature of the medium, and A(κ) is defined as [2,3]:

(5) $$A\left(\kappa \right)={\left(\frac{\left|\kappa \right|M}{\pi k_{B}T}\right)}^{n/2}\left(1+\frac{3\left|\kappa \right|}{2}\right)\frac{\Gamma \left(\frac{1}{2\left|\kappa \right|}+\frac{3}{4}\right)}{\Gamma \left(\frac{1}{2\left|\kappa \right|}-\frac{3}{4}\right)}$$

Two significant advantages of using deformed statistics, such as those of Kaniadakis or Tsallis [4], are the capacity to describe systems with long-term time correlations [5] and the capacity to describe physical phenomena outside thermal equilibrium [3].

Given this considerable versatility, one can find several scientific papers that have applied the Kaniadakis-type deformed statistics. To illustrate this usefulness, one can cite works in astrophysics [6], cosmology [7], DNA analysis [8], quark–gluon plasma [9], game theory [10], error theory [11], information theory [12], random matrices [13], fractal systems [14], dusty-type plasmas [15], gravitational physics [16,17], epidemiology [18], Jeans instability of self-gravitating systems [19], and nuclear reactor physics [3,20,21,22,23].

In the present work, we will review the seven years of application of Kaniadakis statistics in the nuclear reactor physics area, presenting the theories developed until now and reflecting on their future prospects.

## 2. Microscopic Cross Sections and the Doppler Broadening Function

In nuclear reactors, the power generation is guided by neutrons being absorbed by unstable nuclides (uranium, for instance), which, in turn, split into fast-moving lighter nuclides. This fission process generates energy plus additional neutrons, which, again, split other nuclides, starting the nuclear chain reaction.

To understand the neutron–nucleus reaction, it is necessary to present the concept of microscopic cross sections, which are characterized by the probability that a certain reaction will occur and is represented by σ. 

Specifically, from a nuclear reactor physics point of view, one can think in cross sections in the diversity of nuclear reactions, which occur inside a nuclear reactor. These reactions are usually divided in two types. The first one is scattering, $\sigma_{s}$, which has the subdivisions of inelastic scattering, $\sigma_{in}$, and elastic scattering, $\sigma_{e}$. The second division is the absorption types, $\sigma_{a}$, such as radiative capture, $\sigma_{\gamma }$, (n,α) reactions, $\sigma_{\left(n,\alpha \right)}$, and, of course, fission, $\sigma_{f}$. Considering all the possible reactions, one can define a conception of a total microscopic cross section, $\sigma_{t}$, which is, consequently, the sum of all of types and summarizes the probability that any type of neutron–nuclear reaction will occur:

(6) $$\sigma_{t}=\sigma_{s}+\sigma_{a}=\sigma_{e}+\sigma_{in}+\sigma_{f}+\sigma_{\gamma }+\sigma_{\left(n,\alpha \right)}+\ldots$$

The nuclear cross sections will vary strongly according to the incident neutron energy and weakly from the incident beam angle. For this reason, the beam angle is usually ignored in nuclear reactor applications. 

Of all these types of nuclear cross sections, the radiative capture, $\sigma_{\gamma }$, is significantly relevant for nuclear reactor analysis since this is an important factor for removing neutrons from the chain reactions. This process occurs when the incident neutron is absorbed by the nucleus, forming a compound one. After this absorption, this new compound nucleus will decay by the emission of high-energy gamma radiation.

The process of compound nucleus formation occurs only at those energies at which the centre of mass (CM) incident neutron energy plus the neutron binding energy matches the exact energy level of the compound nucleus. Due to this, by adding the Heisenberg’s uncertainty principle considerations, one can obtain a resonance behaviour for the functional dependence of the capture cross sections on the neutron kinetic energy.

In nuclear reactors, neutrons are generated in a highly energetic state in thermal nuclear reactors, far from the ideal energy range to fission U235 nuclei. Consequently, it is necessary to decrease the neutron energies. This process—called neutron slowing down—is conducted by multiple collisions (scattering) in moderators, such as water, graphite, or heavy water. Nevertheless, these scattering processes can lead to absorption, generating undesirable neutron losses.

These absorptions occur in an energy range called the resonance region and are represented by the neutron cross sections. These cross sections, in turn, depend on the relative speed between the neutron and the nuclei. Considering that the nuclei are in thermal motion, the relative velocity can be different (greater or less) than the neutron speed. That fact causes a Doppler effect in the existent neutron cross sections in the resonance region [24]. 

A way to represent this Doppler effect is by utilising the nuclear cross-section through the single-level Breit–Wigner (SLBW) resonance cross section formula. Although there are more modern methodologies, the SLBW method is easier to implement. Besides, it can present analytical functions for reactor physics calculations and, consequently, produce faster processing times [3,25].

One of the cross-section expression components is the Doppler broadening function, which considers a medium in thermal equilibrium at a temperature T. Additionally, the different velocities of the target nuclei are described by the Maxwell–Boltzmann (MB) distribution [26]. The averaged capture resonance cross section formula using the Breit–Wigner methodology represents the thermal nuclei movement and is expressed by [24]:

(7) $${\bar{\sigma }}_{\gamma }=\sigma_{0}\frac{\Gamma_{\gamma }}{\Gamma }{\left(\frac{E_{0}}{E}\right)}^{1/2}\psi \left(x,\xi \right).$$

where:

$\Gamma_{\gamma }/\Gamma \equiv$ the probability that, once formed, the compound nucleus decays to the ground state of the original nucleus by gamma emission;

(8) $$\xi \equiv \frac{\Gamma }{{\left(\frac{4E_{0}k_{B}T}{A}\right)}^{\frac{1}{2}}};$$

(9) $$x\equiv \frac{2}{\Gamma }\left(E-E_{0}\right);$$

A is the mass number, $k_{B}$ is the Boltzmann constant, $\sigma_{0}$ is the value of the total cross section, $\Gamma_{\gamma }$ is radiative line width, Γ is total width of the resonance as measured in the laboratory coordinates, $E_{0}$ is the resonant energy, *E* is the energy of the incident neutron, and ψ(x,ξ) is the so-called Doppler broadening function. 

The Doppler broadening function, after the Bethe and Placzek [27] approximation, can be represented by:

(10) $$\psi \left(\xi ,x\right)\equiv \frac{\xi }{2\sqrt{\pi }}\overset{+\infty }{\underset{-\infty }{\int }}\frac{dy}{1+y^{2}}exp\left[\frac{-\xi^{2}{\left(x-y\right)}^{2}}{4}\right]$$

where:

(11) $$y\equiv \frac{2}{\Gamma }\left(E_{CM}-E_{0}\right)$$

and $E_{CM}$ is the centre-of-mass energy.

**Figure 1:**
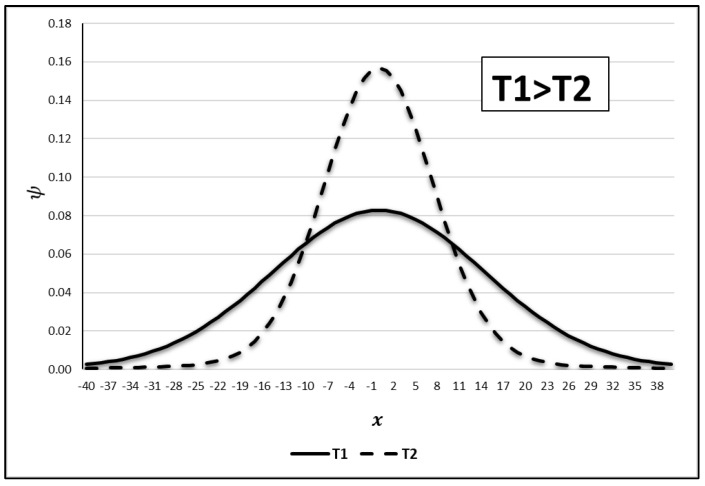
The temperature rise causes the Doppler broadening effect in a resonance curve [28]. Source: de Abreu (2020, p. 14).

Its formulation usually takes into consideration the Maxwell–Boltzmann distribution. By doing a formal analysis, it is possible to note that ψ(ξ,x) is affected by the temperature, i.e., the higher the temperature, the broader the resonance curve, with a peak attenuation, resulting in a higher probability of loss of neutrons by absorption. This effect is represented in Figure 1:

As shown in Figure 1, there is an enlargement in the energy range—illustrated by the x variable—caused by the temperature rise (T1 to T2), which affects the curve’s width. This leads to the probability increase in absorption. 

The Doppler broadening function is also known as the first Voigt function. It represents an even function, i.e., it has symmetry with respect to the energy variable, *x*.

The Doppler broadening phenomenon is of crucial importance for the control of thermal nuclear reactors. This relevance can be observed if one analyzes the expressions for the average cross sections of resonance capture, Equation (7), and scattering, given by:

(12) $${\bar{\sigma }}_{S}=\sigma_{0}\frac{\Gamma_{n}}{\Gamma }\psi \left(\xi ,x\right)+\sigma_{0}\frac{R}{\lambda_{0}}\chi \left(\xi ,x\right)+4\pi R^{2}$$

where:

$\Gamma_{n}/\Gamma \equiv$ the probability that, once formed, the compound nucleus decays to the ground state of the original nucleus by neutron emission;

χ(ξ,x)≡ interference term;

$4\pi R^{2}\equiv$ potential scattering term.

Another way of realizing the importance of the Doppler broadening phenomenon is through the Doppler temperature coefficient. When the fuel temperature of a thermal reactor is increased, there is a growth in the energy range of the nuclear resonances. Hence, there is a decrease in the probability of resonance escape (p), i.e., a reduction in the fraction of fission neutrons moderated to the thermal range without suffering resonant absorption. As the resonance escape probability decreases, the multiplication factor, $\left(k_{eff}\right)$, and, consequently, the reactivity, (ρ), will also be reduced. This relationship is represented in the fuel temperature coefficient—also known as the Doppler temperature coefficient [29]:

(13) $$\alpha_{T_{f}}=\frac{\partial \rho }{\partial T_{f}}=\frac{1}{p}\frac{\partial p}{\partial T_{f}},$$

where $T_{f}$ is the fuel temperature.

### 2.1. Literature Review of Doppler Broadening Functions Approximations Using the Maxwell–Boltzmann Distribution

Over the years, several methods have been developed to obtain approximations of the Doppler broadening function using standard Maxwell–Boltzmann statistics. Therefore, it is relevant to make a brief review of the literature regarding the main methods used, as these methods should also be natural candidates in obtaining generalized solutions.

To conduct this review, we focused on works that considered the approaches proposed by Bethe and Placzek in the year 1936 and which were crucial for the development of the deformed solution for $\psi_{\kappa }\left(\xi ,x\right)$.

#### 2.1.1. Beynon and Grant Methods (1963)

In 1963, T. D. Beynon and I. S. Grant published, in the journal *Nuclear Science and Engineering*, an article that proposed two different methods for calculating the Doppler broadening function and for the interference term (ψ(ξ,x) and χ(ξ,x)), calculated with the aid of a “digital machine”.

The first of these proposed methods use Chebychev polynomial expansions, while the second uses the Gauss–Hermite method. According to the authors, this second method was developed as an alternative to the first due to the excessive memory space spent [30]. Considering that this justification no longer represents a concern for most researchers due to the advancement of technology, the present work will only present the first method, which is more widespread in the literature [3,22,23,25,28,31].

As previously mentioned, the first method proposed by Beynon and Grant, in their 1963 work, carried out the expansion of the exponential part of the integrands of the Doppler broadening function in Chebyshev polynomials. Once this was conducted, the terms were integrated one by one. Before making the expansion, the authors presented the following definitions:

(14) $$a=\frac{\xi }{2}$$

(15) b=ξ·x

Thus, the proposed series are represented as follows:

(16) $$\psi \left(a,b\right)=a\left\{\sqrt{\pi }\cdot cos\left(ab\right)\cdot \left[1-E_{2}\left(a\right)\right]\cdot e^{a^{2}}+J\left(a,b\right)\right\}\cdot e^{-\frac{1}{4}b^{2}}$$

where:

(17) $$\begin{array}{rr} J\left(a,b\right)= & \frac{1}{a}\left\{\frac{1}{2!}{\left(ab\right)}^{2}-\frac{1}{4!}{\left(ab\right)}^{4}+\frac{1}{6!}{\left(ab\right)}^{6}\cdots \right\}+\frac{1}{2a^{3}}\left\{\frac{1}{4!}{\left(ab\right)}^{4}-\frac{1}{6!}{\left(ab\right)}^{6}\cdots \right\}\\ & +\cdots +\frac{1}{\sqrt{\pi }a^{2n+1}}\Gamma \left(\frac{2n+1}{2}\right)\cdot \left\{\frac{1}{\left[2\left(n+1\right)\right]!}{\left(ab\right)}^{2n+1}\cdots \right\}+\cdots \end{array}$$

and

(18) $$E_{2}\left(a\right)=\frac{2}{\sqrt{\pi }}\overset{a}{\underset{0}{\int }}e^{-y^{2}}dy.$$

The Γ(*z*) term represents the gamma function, which is one of the most common special functions in discussions of physical problems. One of the possible ways to express this function is [32]:

(19) $$\Gamma \left(z\right)\equiv \overset{\infty }{\underset{0}{\int }}e^{-t}e^{z-1}dt,\;\mathbb{R}e\left(z\right)>0.$$

Still, in their article, the authors of the work argued that the proposed approximation works well for values where b<6. For values where b≥6, the authors suggested using the asymptotic expansions method [33].

The following method consists of the Taylor series expansion of the term $1/\left(1+y^{2}\right)$ of the Doppler broadening function around y=x, given by [34]:

(20) $$\frac{1}{\left(1+y^{2}\right)}=\frac{1}{\left(1+x^{2}\right)}-\frac{2x}{{\left(1+x^{2}\right)}^{2}}\left(y-x\right)+\frac{-1+3x^{2}}{{\left(1+x^{2}\right)}^{2}}{\left(y-x\right)}^{2}\cdots .$$

By substituting the above-mentioned equation in the approximation proposed by Bethe and Placzek and then performing the integration of all terms, the expansion is obtained:

(21) $$\Psi \left(\xi ,x\right)=\frac{1}{\left(1+x^{2}\right)}\left\{1+\frac{2\left(-1+3x^{2}\right)}{\xi^{2}{\left(1+x^{2}\right)}^{2}}+\frac{12\left(1-10x^{2}+5x^{4}\right)}{\xi^{4}{\left(1+x^{2}\right)}^{4}}\cdots \right\}.$$

For large values of x, Equation (21) tends to:

(22) $$\Psi \left(\xi ,x\right)\approx \frac{1}{\left(1+x^{2}\right)}.$$

Through Equation (21), it is possible to obtain results for the Doppler broadening function considering temperatures close to zero Kelvin [34].

The consideration of alternating methods for different ranges of b (b<6 and b≥6 ) is a strategy that was applied in later works [22,31]. 

The tables of values for the Doppler broadening function and for the interference term generated by the work of Beynon and Grant were essential for the reactor physics field, being present even in two of the main books in this field [24,29].

#### 2.1.2. Campos and Martinez Method (1987)

In 1987, Campos and Martinez published, in the journal *Annals of Nuclear Energy*, the article “The dependence of practical width on temperature”. This work, among other topics, presented a new procedure for calculating the Doppler broadening function. To do that, the authors used the definitions of Beynon and Grant for the first Voigt function, ψ(ξ,x), and the interference term, χ(ξ,x), also known as second Voigt function.

For this, the authors differentiated both the integral definition of the Doppler broadening function and the interference term in relation to x, arriving at the following differential equation [31]:

(23) $$\frac{4}{\xi^{2}}\frac{\partial^{2}\psi \left(\xi ,x\right)}{\partial x^{2}}+4x\frac{\partial \psi \left(\xi ,x\right)}{\partial x}+\frac{\xi^{2}}{4}\left[2+\xi^{2}x^{2}+\xi^{2}\right]\psi \left(\xi ,x\right)=\xi^{2}$$

subject to the following initial conditions:

(24) $${\psi \left(\xi ,x\right)|}_{x=0}=\psi_{0}=\frac{\xi \sqrt{\pi }}{2}e^{\frac{\xi^{2}}{4}}\left[1-\mathrm{erf}\left(\frac{\xi }{2}\right)\right]$$

(25) $${\frac{\partial \psi \left(\xi ,x\right)}{\partial x}|}_{x=0}=0.$$

To solve the proposed differential equation, Campos and Martinez (1987) used the power series expansion method, presenting the following result:

(26) $$\psi \left(\xi ,x\right)=\overset{\infty }{\underset{n=0}{\sum }}c_{n}x^{2n},$$

with the coefficients generated from the recurrence formulas:

(27) $$c_{0}=\psi_{0};$$

(28) $$c_{1}=\frac{\xi^{2}}{8}\left[\xi^{2}-\left(\xi^{2}+2\right)\psi_{0}\right];$$

(29) $$c_{n+1}=-\frac{\xi^{2}}{4}\left[\frac{\left(4n+\xi^{2}+2\right)c_{n}+\xi^{2}c_{n-1}}{\left(n+2\right)\left(n+1\right)}\right]$$

Similarly to Beynon and Grant, Campos and Martinez also used the alternation of methods for different ranges of b (b<6 and b≥6), using the same Taylor expansion method, demonstrated by Equation (21) for values of b≥6.

Additionally, in this 1987 work, an expression for the temperature-dependent practical width was developed, which, in turn, was used later in developing the Campos–Martinez model for resonance integrals for isolated ones [31,35].

#### 2.1.3. The Palma, Martinez, and Silva Method (2006)

In 2006, Palma, Martinez, and Silva published, in the *Journal of Nuclear Science and Technology*, the article “The derivation of the Doppler broadening function using Frobenius method” [36].

In this work, the authors solved Equation (23) using a different methodology from the one proposed by Campos and Martinez: utilizing the Frobenius method for the resolution of the homogeneous part of the equation and the method of variation of parameters to obtain the particular solution.

Taking this into account, the solution for the obtained Doppler broadening function was:

(30) $$\psi \left(\xi ,x\right)=\frac{\xi \sqrt{\pi }}{2}e^{-\frac{\xi^{2}}{4}\left(x^{2}-1\right)}cos\left(\frac{\xi^{2}x}{2}\right)\left[1+Re\phi \left(\xi ,x\right)+tan\left(\frac{\xi^{2}x}{2}\right)Im\phi \left(\xi ,x\right)\right]$$

where

(31) $$\phi \left(\xi ,x\right)=erf\left(\frac{i\xi x-\xi }{2}\right)$$

To measure the method’s accuracy, the authors compared the results of Equation (30) with results obtained from Padé’s four-pole method [37,38], which is widely used in calculating the function of Doppler broadening and for calculating resonance integrals. According to Palma et al., approximations of this type are better than using the Taylor series to describe functions with poles.

This comparison concluded that the proposed solution presented relative percentage errors lower than the Padé method [36].

## 3. Kaniadakis Entropy in the Context of Nuclear Reactor Physics

In the context of the current thermal nuclear reactors scenario, it is appropriate to apply the Maxwell–Boltzmann statistics to treat the nuclei velocities distribution. However, considering the perspective of a new generation of nuclear reactors with different characteristics, the MB approach will not be enough, especially if one considers describing situations outside the thermal equilibrium [3].

Guedes et al. [3] proposed using the Kaniadakis statistics to obtain an expression for the Doppler broadening function, which could be capable of contemplating situations other than the regular nuclear reactors, for instance, in the context of thermal non-equilibrium. 

To do this, the authors proposed a new integral expression, $\psi_{\kappa }\left(\xi ,x\right)$, starting from the Doppler broadening function without any approximations, Ψ(ξ,x), given by [3]:

(32) $$\Psi \left(\xi ,x\right)\equiv \pi \sqrt{\frac{2k_{B}T}{M}}\xi \overset{+\infty }{\underset{-\frac{2}{\Gamma }E_{O}}{\int }}\frac{dy}{1+y^{2}}\int_{v\left(x\right)-v_{r}\left(y\right)}^{v\left(x\right)+v_{r}\left(y\right)}dVVf\left(V,T\right),$$

where v is the neutron velocity, $v_{r}$ the relative velocity between the neutron and the nucleus, and f(V,T) is the chosen nuclei velocities distribution. By substituting the Kaniadakis statistics, $f_{\kappa }\left(V,T\right)$, into the equation above, one has [3]:

(33) $$\Psi \left(\xi ,x\right)\equiv \pi \sqrt{\frac{2k_{B}T}{M}}A\left(\kappa \right)\xi \overset{+\infty }{\underset{-\frac{2}{\Gamma }E_{O}}{\int }}\frac{dy}{1+y^{2}}\int_{v\left(x\right)-v_{r}\left(y\right)}^{v\left(x\right)+v_{r}\left(y\right)}dVV\exp_{\kappa }\left(-\frac{MV^{2}}{2k_{B}T}\right).$$

After some algebraic manipulation to perform the integration of the *V* variable, Guedes et al. [3] presented a new function, called $iexp_{\kappa }$, since:

(34) $$\int \exp_{\kappa }\left(x\right)dx=i\exp_{\kappa }\left(x\right)+C,$$

where

(35) $$i\exp_{\kappa }\left(x\right)\equiv \left(\frac{\sqrt{1+\kappa^{2}x^{2}}-\kappa^{2}x}{1-\kappa^{2}}\right)\exp_{\kappa }\left(x\right);$$

Noting that:

(36) $$\underset{\kappa \to 0}{\lim}i\exp_{\kappa }\left(x\right)=e^{x},$$

Equation (33) becomes:

(37) $$\Psi \left(\xi ,x\right)\equiv \frac{\xi }{2\sqrt{\pi }}B\left(\kappa \right)\overset{+\infty }{\underset{-\frac{2}{\Gamma }E_{O}}{\int }}\frac{dy}{1+y^{2}}\left[i\exp_{\kappa }\left(-\frac{1}{2k_{B}T}M{\left(v\left(x\right)-v_{r}\left(y\right)\right)}^{2}\right)-i\exp_{\kappa }\left(-\frac{1}{2k_{B}T}M{\left(v\left(x\right)+v_{r}\left(y\right)\right)}^{2}\right)\right].$$

where:

(38) $$B\left(\kappa \right)={\left(2\left|\kappa \right|\right)}^{3/2}\left(1+\frac{3\left|\kappa \right|}{2}\right)\frac{\Gamma \left(\frac{1}{2\left|\kappa \right|}+\frac{3}{4}\right)}{\Gamma \left(\frac{1}{2\left|\kappa \right|}-\frac{3}{4}\right)}.$$

Finally, by using the Bethe–Placzek [27] approximations, Guedes et al. provided the integral solution for the Doppler broadening function using the Kaniadakis distribution:

(39) $$\Psi_{\kappa }\left(\xi ,x\right)\approx \psi_{\kappa }\left(\xi ,x\right)=\frac{\xi }{2\sqrt{\pi }}B\left(\kappa \right)\overset{+\infty }{\underset{-\infty }{\int }}\frac{dy}{1+y^{2}}i\exp_{\kappa }\left[\frac{-\xi^{2}{\left(x-y\right)}^{2}}{4}\right].$$

Noting again that, when κ tends towards zero, one can obtain the integral solution for the Doppler broadening function using the Maxwell–Boltzmann distribution.

**Figure 2:**
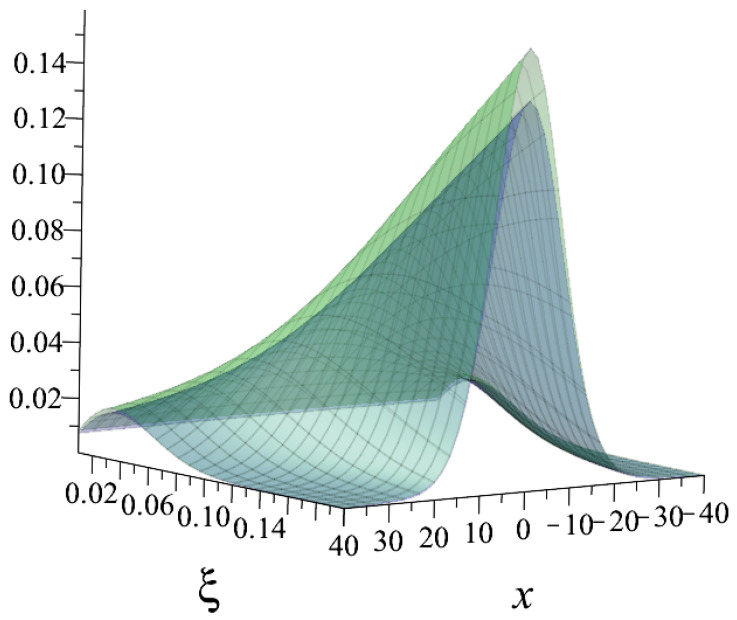
The comparison between the $\psi_{\kappa =0.3}\left(\xi ,x\right)$ (blue) and ψ(ξ,x) (green) curves [28]. It is possible to see the peak’s attenuation.

**Figure 3:**
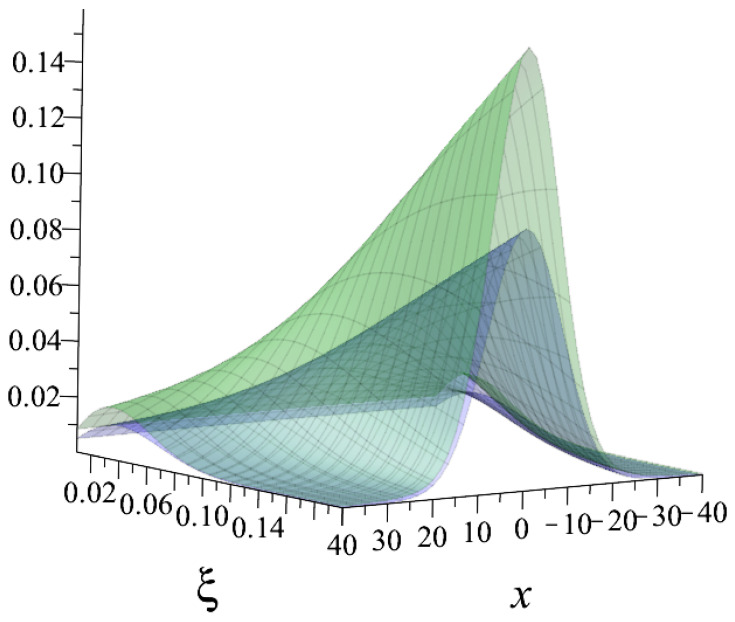
The comparison between the $\psi_{\kappa =0.5}\left(\xi ,x\right)$ (blue) and ψ(ξ,x) (green) curves. It is possible to see the peak’s attenuation.

The primary behaviour with the usage of the Kaniadakis statistical distribution is the peak attenuation for the resonance curves as the value of κ rises, as one can see in the Figure 2 and Figure 3 below:

An animation of this behaviour for different values of κ is available at the following link: “https://youtu.be/wAeBAETbU1c (Accessed on 1 March 2023)”. 

## 4. Analytical Solutions for the Doppler Broadening Function Using the Kaniadakis Distribution

Guedes et al. [3] presented the results of the integral formulation for the Doppler broadening function using the Kaniadakis distribution, Equation (39), by using a numerical calculation, which, in turn, can present very high processing times when inserted into complex systems of calculations, such as nuclear reactor simulations.

In the already existing computer codes for nuclear core design, the Doppler broadening process requires a relatively long computing time to calculate Equation (10). Therefore, more time will be needed to calculate the deformed function, Equation (39). One should keep in mind that the Doppler broadening function for neutron cross sections should be calculated for each energy mesh point, isotope, and temperature.

In order to surpass this potential problem, de Abreu et al. proposed an analytical solution for the integral expression. To do that, the authors started by obtaining a differential equation for $\psi_{\kappa }\left(\xi ,x\right)$ from the integral expression, using the same methodology by Campos and Martinez for obtaining an analytical expression for ψ considering the Maxwell–Boltzmann distribution.

(40) $$\frac{\partial^{2}\psi_{\kappa }\left(\xi ,x\right)}{\partial x^{2}}-\frac{\xi^{2}x}{2}\left[\frac{{\left(\kappa^{2}-1\;\right)}^{2}+1}{\left(\kappa^{2}-1\;\right)}\right]\frac{\partial \psi_{\kappa }\left(\xi ,x\right)}{\partial x}+\frac{\xi^{2}}{4}\left[-2\left(\kappa^{2}-1\;\right)+\xi^{2}x^{2}+\xi^{2}\right]\psi_{\kappa }\left(\xi ,x\right)=-\frac{\xi^{4}}{4}\left(\kappa^{2}-1\;\right)B\left(\kappa \right).$$

One relevant thing to mention is that when κ tends to zero, the differential equation proposed by Abreu et al. [21,22] becomes the equation proposed by Campos and Martinez, proving the validity of the solution.

Initially, to solve the deformed differential equation using the Kaniadakis distribution, the authors used the same method by Palma, Martinez, and Gonçalves, i.e., the Frobenius method, for the homogeneous part and the parameter variation method for the particular solution. However, to eliminate some of the approximations and assumptions made, a new paper was published later using the dependent variable method to solve the homogeneous part of the equation. 

The solution of the deformed differential equation for $\psi_{\kappa }$ is divided in two based on the above-mentioned works of Beynon and Grant [30] and Campos and Martinez [31]: one for |x·ξ|<6 and other for |x·ξ|≥6. The first one can be represented by:

(41) $$\psi_{k}\left(\xi ,x\right)=\Lambda \left(x,\xi \right)\left[D\left(\xi ,x\right)+\Omega_{g}\left(\xi ,x\right)\right],$$

where

(42) D(ξ,x)≡[Δ(ξ)·cos(Θ)],

(43) $$\Omega_{g}\left(\xi ,x\right)\equiv \Pi \left(x,\xi \right)\cdot \left[i\Omega_{1}\left(\xi ,x\right)+\Omega_{2}\left(\xi ,x\right)\right];$$

(44) $$\Lambda \left(\xi ,x\right)=\exp\left(\frac{\xi^{2}-\xi^{2}x^{2}}{4}\right)\cdot \frac{\xi \sqrt{\pi }B\left(\kappa \right)}{4};$$

(45) $$\Pi \left(\xi ,x\right)=\frac{\sqrt{\xi^{4}-2\xi^{2}\kappa^{2}}}{-\xi^{2}+2\kappa^{2}}\cdot \exp\left(\frac{-\kappa^{2}}{2}\right);$$

(46) Δ(ξ)=2−2erf(ξ2)1−κ2.

(47) $$\Omega_{1}\left(\xi ,x\right)=sin\left(\Theta \right)\cdot \left[erf\left(P_{1}\right)\kappa^{2}-erf\left(P_{1}\right)+erf\left(P_{2}\right)\kappa^{2}-erf\left(P_{2}\right)\right];$$

(48) $$\Omega_{2}\left(\xi ,x\right)=cos\left(\Theta \right)\cdot \left[2erf\left(P_{3}\right)\kappa^{2}-2erf\left(P_{3}\right)-erf\left(P_{1}\right)\kappa^{2}+erf\left(P_{1}\right)+erf\left(P_{2}\right)\kappa^{2}-erf\left(P_{2}\right)\right];$$

(49) $$P_{1}\left(\xi ,x\right)=\frac{-i\xi^{2}x+\sqrt{\xi^{4}-2\xi^{2}\kappa^{2}}}{2\xi };$$

(50) $$P_{2}\left(\xi ,x\right)=\frac{-i\xi^{2}x-\sqrt{\xi^{4}-2\xi^{2}\kappa^{2}}}{2\xi };$$

(51) $$P_{3}\left(\xi ,x\right)=\frac{\sqrt{\xi^{4}-2\xi^{2}\kappa^{2}}}{2\xi };$$

(52) $$\Theta \left(\xi ,x\right)=\frac{x}{2}\sqrt{\xi^{4}-2\xi^{2}\kappa^{2}};$$

The solution for the |x·ξ|≥6 domain is obtained by the conduction of asymptotic expansions in the Taylor series. Thus, the expression for $\psi_{\kappa }\left(\xi ,x\right)$ in this case is:

(53) $$\psi_{\kappa }⟦\left|x\cdot \xi \right|\geq 6⟧≅B\left(\kappa \right)\cdot \left[\frac{1}{\left(1-\kappa^{2}\right)\left(1+x^{2}\right)}+\frac{-3\kappa^{2}\xi^{2}-\kappa^{2}\xi^{2}x^{4}+4-12x^{2}}{2\xi^{2}\left(\kappa^{2}-1\right){\left(1+x^{2}\right)}^{3}}+\ldots \right]$$

In a more recent paper [25], the authors also calculated the computational processing times. They obtained an average value of 4.6 for the ratio (numerical/analytical), confirming the predicted faster processing times for the analytical solution.

In parallel with the development of analytical solutions, da Silva et al. [39] proposed a method to establish a relation of equivalence among the standard Doppler broadening function, ψ(ξ,x), and the deformed one, $\psi_{\kappa }\left(\xi ,x\right)$. 

The effective medium temperature model consists of determining the temperature, $T_{eff}$, which will have the Doppler broadening function from the perspective of the Maxwell–Boltzmann distribution reproducing the same value for the function using Kaniadakis distribution in the actual temperature (T) of the medium, that is:

(54) $$\psi_{\kappa }\left(x,\xi \right)≅\;\psi_{\kappa }\left(x,\tilde{\xi }\right)$$

where:

(55) $$\tilde{\xi }\equiv \frac{\Gamma }{{\left(4E_{0\;}k_{B}T_{eff}/A\right)}^{1/2}}$$

and

(56) $$\xi \equiv \frac{\Gamma }{{\left(4E_{0\;}k_{B}T/A\right)}^{1/2}}$$

Applying the polynomial regression method optimised by the genetic algorithm technique, the values of $\tilde{\xi }$ and, consequently, of the effective medium temperature, $T_{eff}\;$, were obtained.

Thus, the deformed Doppler broadening function, $\psi_{\kappa }\left(x,\xi \right)$, according to the Kaniadakis distribution, can be obtained directly via the original Doppler broadening function by replacing the medium temperature, T, with the effective temperature of the medium, $T_{eff}$.

According to the authors, the model proved to be a much faster and simpler method for calculating deformed Doppler broadening functions concerning the numerical approach [39].

In this ‘twenty years of Kaniadakis entropy’ issue, a paper was published [23], applying the analytical solution for $\psi_{k}$ to calculate deformed nuclear cross sections in the nuclear data processing code FRENDY, developed by the Japan Atomic Energy Agency.

One of the contributions of this work was to study the relevance of the so-called Gaussian error function, erf, in the proposed analytical formulation for $\psi_{k}$. The erf function is defined by:

(57) $$erf\left(x\right)=\frac{2}{\sqrt{\pi }}\int_{0}^{x}e^{-t^{2}}dt.$$

Due to its complexity, many programming code libraries use approximations to calculate these functions. These approximations, in turn, result in errors. The authors then used the Faddeeva Package [28] to minimise these, especially for the tails (far from the resonance peaks) regions.

## 5. Kaniadakis’ Entropy Validation in the Nuclear Reactor Physics

Other areas that employed the Kaniadakis statistics can validate its results through observational data (stellar clusters, for instance). For an example of this validation, one can present the high-quality agreement between the theoretical curves for cosmic rays’ flux using the Kaniadakis distribution and observational data. As one can see in reference [2], the Kaniadakis statistics represented the observational data better than the standard Maxwell-Boltzmann one.

However, in nuclear physics reactors, this is not possible. Thus, one needs to find ways to validate the applicability of this generalised entropy in the nuclear engineering area. 

From this perspective, it is crucial—from a validation point of view—to verify the existence of a pertinency in applying the Kaniadakis method in the reactor physics area. Hence, one of the first necessary steps is to generate deformed nuclear data and compare it to the Maxwell–Boltzmann results. 

Nevertheless, before this step, it was necessary to validate the analytical solution itself. Hence, in a 2020 work, de Abreu [28] generated the first deformed data using the following equations for the deformed radiative capture cross sections obtained through the Doppler broadening function considering the Kaniadakis distribution:

(58) $$\sigma_{\gamma }^{\kappa }=\sigma_{0}\left(\frac{\Gamma_{\gamma }}{\Gamma }\right){\left(\frac{E_{0}}{E}\right)}^{\frac{1}{2}}\psi_{\kappa }\left(\xi ,x\right)$$

**Figure 4:**
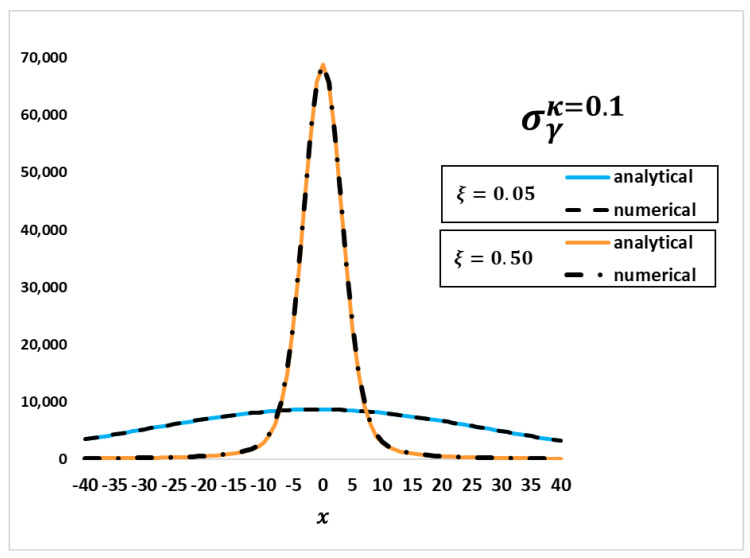
A comparison between the analytical and numerical results for the radiative capture cross-sections using the Kaniadakis distribution [28]. Source: de Abreu (2020, p. 90, our translation).

To calculate these cross sections, the authors used the parameters — illustrated in Table 1—of the 238 isotope of uranium in the 6.67 eV peak resonance:

Using the above-mentioned data and Equation (58), the authors generated the deformed analytical data by using the numerical and analytical expressions for $\psi_{\kappa }\left(\xi ,x\right)$ to compare them with each other, as shown in Figure 4. 

The results show a robust agreement between both solutions, which gives conditions to proceed in the validation steps. 

However, even with the results in conformity with the predicted behaviour concerning the numerical results, applying the Kaniadakis distribution in a proper nuclear data generator tool is still necessary for certification purposes. In this sense, one needs to insert the analytical solution into nuclear data processing codes, preferably into ones recognised and used by peers around the world, for instance, FRENDY [28,29], NJOY [40], PREPRO [41], or NECP-Atlas [42]. The advantage of using these systems is that they can process official evaluated nuclear data libraries, such as ENDF, CENDL, JEFF, or JENDL. 

**Figure 5:**
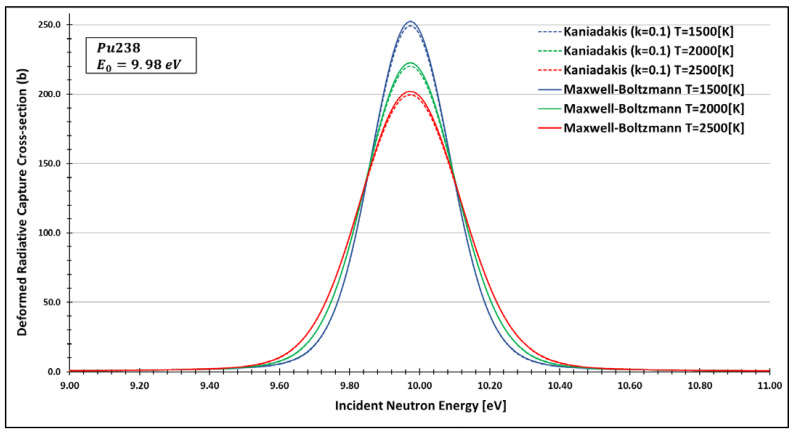
A plot of the deformed radiative capture cross section for plutonium 238 considering k=0.1 and the 9.98 eV peak generated by the FRENDY data processing code [23]. Source: de Abreu et al. (2022, p. 10).

**Figure 6:**
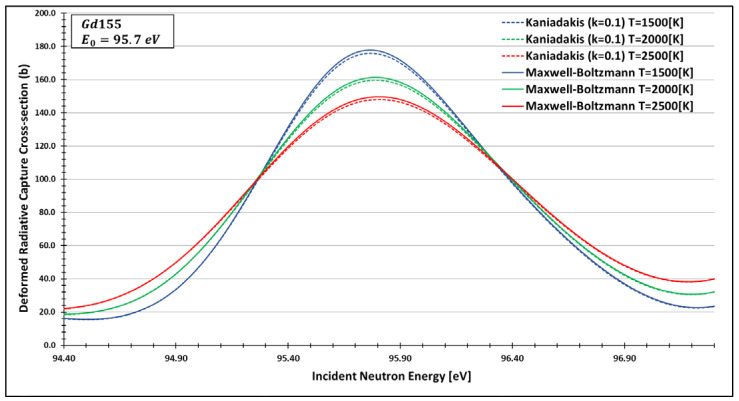
A plot of the deformed radiative capture cross section for gadolinium 155 considering k=0.1 and the 95.7 eV peak generated by the FRENDY data processing code [23]. Source: de Abreu et al. (2022, p. 12).

Therefore, a paper in this twenty-year commemorative issue was published, generating, for the first time, deformed data using the Kaniadakis distribution for two important nuclides: technetium 99 and plutonium 238, as one can see in Figure 5 and Figure 6. To do that, they chose the FRENDY nuclear data processing code [28,29].

By analysing the data, the authors found compatibility between the expected peak attenuation behaviour of the deformed curves compared to the Maxwell–Boltzmann data for different resonance peaks. 

These results represent a significant step in validating the Kaniadakis statistics in the nuclear fission area. 

## 6. Next Steps and Perspectives of Kaniadakis Entropy in the Nuclear Reactor Physics

Much work has been conducted on nuclear reactor physics with the Kaniadakis deformed statistics. However, to proceed with this investigation, there is still a question to answer: which value (or range) of κ fits with current and future nuclear reactors? 

In this sense, future works will need to proceed with the work inside data generation codes and use its results of deformed nuclear cross sections in other nuclear reactor simulations to find the closest values of the deformation factor, κ, in order to fit the standard nuclear reactor behaviour.

Another possible and relevant step is implementing the Kaniadakis methodology in modern theoretical methods, such as the multi-level Breit–Wigner and Reich–Moore methods. With this implementation, it would be possible, for instance, to generate a deformed nuclear cross section for other nuclides, such as the isotopes 235 and 238 of uranium.

## Figures and Tables

**Table 1:** Resonance data for the 6.67 eV line.

$E_{0}\left(\mathrm{eV}\right)$	$\Gamma_{n}\left(\mathrm{eV}\right)$	$\Gamma_{\gamma }\left(\mathrm{eV}\right)$	$\sigma_{0}\;\left(b\right)$	$\Gamma_{p}\left(\mathrm{eV}\right)$
6.67	0.00152	0.026	$2.16\times 10^{5}$	1.26

Source: Duderstadt and Hamilton [24] (1976, p. 335).

## Data Availability

Not applicable.

## References

[1] Kaniadakis G. (2001). Non-Linear Kinetics Underlying Generalized Statistics. Phys. A Stat. Mech. Its Appl..

[2] Kaniadakis G. (2002). Statistical Mechanics in the Context of Special Relativity. Phys. Rev. E.

[3] Guedes G., Gonçalves A.C., Palma D.A. (2017). The Doppler Broadening Function Using the Kaniadakis Distribution. Ann. Nucl. Energy.

[4] Tsallis C. (1988). Possible Generalization of Boltzmann-Gibbs Statistics. J. Stat. Phys..

[5] Chavanis P.-H. (2003). Generalized Thermodynamics and Fokker-Planck Equations: Applications to Stellar Dynamics and Two-Dimensional Turbulence. Phys. Rev. E.

[6] Carvalho J.C., Silva R., do Nascimento J.D., Soares B.B., De Medeiros J.R. (2010). Observational Measurement of Open Stellar Clusters: A Test of Kaniadakis and Tsallis Statistics. EPL Europhys. Lett..

[7] Luciano G.G. (2022). Gravity and Cosmology in Kaniadakis Statistics: Current Status and Future Challenges. Entropy.

[8] de Lima M.M.F., Anselmo D.H.A.L., Silva R., Nunes G.H.S., Fulco U.L., Vasconcelos M.S., Mello V.D. (2022). A Bayesian Analysis of Plant DNA Length Distribution via κ-Statistics. Entropy.

[9] Teweldeberhan A.M., Miller H.G., Tegen R. (2003). κ-deformed statistics and the formation of a quark-gluon plasma. Int. J. Mod. Phys. E.

[10] Topsøe F. (2004). Entropy and Equilibrium via Games of Complexity. Phys. A Stat. Mech. Its Appl..

[11] Wada T., Suyari H. (2006). κ-Generalization of Gauss’ Law of Error. Phys. Lett. A.

[12] Wada T., Suyari H. (2007). A Two-Parameter Generalization of Shannon–Khinchin Axioms and the Uniqueness Theorem. Phys. Lett. A.

[13] Abul-Magd A.Y. (2007). Nonextensive Random-Matrix Theory Based on Kaniadakis Entropy. Phys. Lett. A.

[14] Olemskoi A.I., Kharchenko V.O., Borisyuk V.N. (2008). Multifractal Spectrum of Phase Space Related to Generalized Thermostatistics. Phys. A Stat. Mech. Its Appl..

[15] Lourek I., Tribeche M. (2019). Dust Charging Current in Non Equilibrium Dusty Plasma in the Context of Kaniadakis Generalization. Phys. A Stat. Mech. Its Appl..

[16] Abreu E.M.C., Neto J.A., Barboza E.M., Nunes R.C. (2017). Tsallis and Kaniadakis Statistics from the Viewpoint of Entropic Gravity Formalism. Int. J. Mod. Phys. A.

[17] Abreu E.M.C., Neto J.A., Mendes A.C.R., Bonilla A., de Paula R.M. (2018). Cosmological Considerations in Kaniadakis Statistics. EPL Europhys. Lett..

[18] Kaniadakis G., Baldi M.M., Deisboeck T.S., Grisolia G., Hristopulos D.T., Scarfone A.M., Sparavigna A., Wada T., Lucia U. (2020). The κ-Statistics Approach to Epidemiology. Sci. Rep..

[19] He K.-R. (2022). Jeans Analysis with κ-Deformed Kaniadakis Distribution in f (R) Gravity. Phys. Scr..

[20] de Abreu W.V., Gonçalves A.C., Martinez A.S. (2019). An Analytical Approximation for the Generalized Interference Term Using the Kaniadakis Distribution. Proc. Int. Conf. Nucl. Eng..

[21] de Abreu W.V., Gonçalves A.C., Martinez A.S. (2019). Analytical Solution for the Doppler Broadening Function Using the Kaniadakis Distribution. Ann. Nucl. Energy.

[22] de Abreu W.V., Gonçalves A.C., Martinez A.S. (2020). New Analytical Formulations for the Doppler Broadening Function and Interference Term Based on Kaniadakis Distributions. Ann. Nucl. Energy.

[23] de Abreu W.V., Maciel J.M., Martinez A.S., da Cruz Gonçalves A., Schmidt L. (2022). Doppler Broadening of Neutron Cross-Sections Using Kaniadakis Entropy. Entropy.

[24] Duderstadt J.J., Hamilton L.J. (1976). Nuclear Reactor Analysis.

[25] de Abreu W.V., Martinez A.S., do Carmo E.D., Gonçalves A.C. (2021). A Novel Analytical Solution of the Deformed Doppler Broadening Function Using the Kaniadakis Distribution and the Comparison of Computational Efficiencies with the Numerical Solution. Nucl. Eng. Technol..

[26] Pathria R.K., Beale P.D. (2011). Statistical Mechanics.

[27] Bethe H.A., Placzek G. (1937). Resonance Effects in Nuclear Processes. Phys. Rev..

[28] de Abreu W.V. (2020). de Solução Analítica Da Função de Alargamento Doppler Usando a Distribuição de Kaniadakis. Ph.D. Thesis (Nuclear Engineering).

[29] Stacey W.M. (2007). Nuclear Reactor Physics.

[30] Beynon T.D., Grant I.S. (1963). Evaluation of the Doppler-Broadened Single-Level and Interference Functions. Nucl. Sci. Eng..

[31] Campos T.P.R., Martinez A.S. (1987). The Dependence of Practical Width on Temperature. Ann. Nucl. Energy.

[32] Arfken G.B., Weber H.J., Harris F.H. (2013). Mathematical Methods for Physicists.

[33] Melkonian E., Havens W.W., Rainwater L.J. (1953). Slow Neutron Velocity Spectrometer Studies. V. Re, Ta, Ru, Cr, Ga. Phys. Rev..

[34] Gonçalves A. (2010). da C. Cálculo Da Função de Alargamento Doppler Utilizando Análise de Fourier. Ph.D. Thesis (Nuclear Engineering).

[35] Marguet S. (2017). The Physics of Nuclear Reactors.

[36] Palma D.A., Martinez A.S., Silva F.C. (2006). The Derivation of the Doppler Broadening Function Using Frobenius Method. J. Nucl. Sci. Technol..

[37] Keshavamurthy R.S., Harish R. (1993). Use of Padé Approximations in the Analytical Evaluation of the J (θ,β) Function and Its Temperature Derivative. Nucl. Sci. Eng..

[38] Shcherbakov O., Harada H. (2002). Resonance Self-Shielding Corrections for Activation Cross Section Measurements. J. Nucl. Sci. Technol..

[39] da Silva M.V., Martinez A.S., Gonçalves A.C. (2021). Effective Medium Temperature for Calculating the Doppler Broadening Function Using Kaniadakis Distribution. Ann. Nucl. Energy.

[40] Macfarlane R., Muir D.W., Boicourt R.M., Kahler I.A.C., Conlin J.L. (2017). The NJOY Nuclear Data Process. System, Version 2016.

[41] (2010). International Atomic Energy Agency PREPRO. https://www-nds.iaea.org/public/endf/prepro/.

[42] Zu T., Xu J., Tang Y., Bi H., Zhao F., Cao L., Wu H. (2019). NECP-Atlas: A New Nuclear Data Processing Code. Ann. Nucl. Energy.

